# Integration of the PortionSize Ed App into SNAP-Ed for Improving Diet Quality Among Adolescents in Hawaii: A Randomized Pilot Study

**DOI:** 10.3390/nu17193145

**Published:** 2025-10-01

**Authors:** Emerald S. Proctor, Kiari H. L. Aveiro, Ian Pagano, Lynne R. Wilkens, Leihua Park, Leilani Spencer, Jeannie Butel, Corby K. Martin, John W. Apolzan, Rachel Novotny, John Kearney, Chloe P. Lozano

**Affiliations:** 1 School of Biological, Health and Sports Sciences, Technological University Dublin, D07 H6K8 Dublin, Irelandjohn.kearney@tudublin.ie (J.K.); 2 School of Medicine, Trinity College Dublin, D02 PN40 Dublin, Ireland; 3 University of Hawaii Cancer Center, University of Hawaii at Mānoa, Honolulu, HI 96813, USA; 4 University of Hawaii at Manoa, Honolulu, HI 96813, USAnovotny@hawaii.edu (R.N.); 5 Pennington Biomedical Research Center, Baton Rouge, LA 70808, USA; corby.martin@pbrc.edu (C.K.M.); john.apolzan@pbrc.edu (J.W.A.)

**Keywords:** behavior change, dietary assessment, food photography, mHealth, MyPlate, adolescent nutrition, nutrition education, eating patterns, SNAP-Ed, PortionSize Ed

## Abstract

**Background/Objectives:** Coupling mobile health (mHealth) technology with community-based nutrition programs may enhance diet quality in adolescents. This pilot study evaluated the feasibility, acceptability, and preliminary efficacy of integrating PortionSize Ed (PSEd), an image-assisted dietary assessment and education app, into the six-week Hawaii Food and Lifeskills for Youth (HI-FLY) curriculum delivered via Supplemental Nutrition Assistance Program Education (SNAP-Ed). **Methods:** Adolescents (grades 6–8) from two classrooms were cluster-randomized into HI-FLY or HI-FLY + PSEd. Both groups received HI-FLY and completed Youth Questionnaires (YQ) and food records (written or app-based) at Weeks 0 and 7. Feasibility and acceptability were assessed via enrollment, attrition, and User Satisfaction Surveys (USS). Diet quality was measured using Healthy Eating Index-2020 (HEI-2020) scores and analyzed via mixed-effects models. **Results:** Of 50 students, 42 (84%) enrolled and attrition was minimal (2.4%). The sample was 49% female and 85% at least part Native Hawaiian or Pacific Islander (NHPI). PSEd was acceptable, with average USS scores above the scale midpoint. No significant HEI-2020 changes were observed, though YQ responses indicated improvements in sugary drink intake (*p* = 0.03) and use of nutrition labels in HI-FLY + PSEd (*p* = 0.0007). **Conclusions:** Integrating PSEd into SNAP-Ed was feasible, acceptable, and demonstrated potential healthy behavior change among predominantly NHPI youth in Hawaii.

## 1. Introduction

Childhood obesity is a major global health concern, linked to an elevated risk of non-communicable diseases, reduced quality of life, and substantial healthcare costs [1]. In the United States (US), 17% of children aged 6–17 years in the US were living with obesity in 2022–2023 [2], with disproportionately greater rates observed in low-income and minority groups [3,4]. Native Hawaiian and Pacific Islander (NHPI) youth experience disproportionately high obesity rates, with prevalence exceeding 20% among Native Hawaiian children and over 40% among other Pacific Islander youth [2,5]. This largely driven by socio-economic and environmental factors [6,7]. These disparities highlight the urgent need culturally relevant, community-driven interventions tailored to the unique context of Pacific populations [8,9]. Early prevention is critical, given that childhood obesity is associated with later-life conditions such as adult obesity, metabolic disorders, certain cancers, and mental health conditions [10,11].

Supplemental Nutrition Assistance Program Education (SNAP-Ed) is a federally funded public health initiative, promoting healthy eating and physical activity in alignment with the Dietary Guidelines for Americans (DGA) among US populations with low income [12]. In Hawaii, SNAP-Ed is delivered through the Hawaii Food and Life Skills for Youth (HI-FLY) program, a culturally adapted school-based curriculum designed to promote nutrition literacy and healthy eating among adolescents [13]. A recent cross-sectional analysis highlighted the program’s efficacy in Hawaii, showing that NHPI adolescents from SNAP-participating families had 77% lower odds of obesity, compared to non-participant [6]. However, current program evaluation methods rely on self-reported dietary behaviors, which are prone to bias and inaccuracies [14]. There is a need for more accurate and engaging approaches to dietary assessment that can also reinforce nutrition education among adolescents.

Mobile health (mHealth) technologies offer promising solutions for real-time dietary assessment and behavior change. Mobile apps are increasingly accessible across income levels and may enhance youth engagement and accuracy in dietary reporting [15]. While initial studies suggest that mHealth tools can promote healthier choices among youth, evidence remains limited, particularly among underserved adolescent populations [4,16,17,18,19]. Furthermore, the integration of culturally tailored mHealth tools into community-based nutrition programs remains unexplored among NHPI youth.

To address this gap, PortionSize Ed (PSEd), a culturally tailored, image-assisted dietary assessment and education app, was developed in collaboration with SNAP-Ed Hawaii [20,21]. PSEd aligns with the Dietary Guidelines for Americans and MyPlate recommendations and provides real-time feedback on food group intake using image-assisted food records [22,23]. The app-based food records can then be used to assess diet quality as per Healthy Eating Index-2020 (HEI-2020) scores [24]. By combining digital technology with community-based education models like SNAP-Ed, PSEd has the potential to modernize dietary assessment and enhance nutrition education in a culturally relevant manner.

The primary aim of this pilot study was to assess the feasibility and acceptability of integrating the PSEd mobile app [Version 1.0 (103)] into SNAP-Ed among middle school students in Hawaii. The secondary aim was to investigate the preliminary efficacy of using PSEd alongside SNAP-Ed to improve diet quality among this cohort, as measured by the HEI-2020.

## 2. Materials and Methods

### 2.1. Ethical Approval

Ethical approval was granted for this pilot from the Institutional Review Board at the University of Hawaii (IRB Federal Wide Assurance number: 2023-00060). This study was conducted in accordance with the Declaration of Helsinki [25].

### 2.2. Study Design

A 7-week parallel cluster-randomized trial design was employed across two classrooms in a middle school in Hawaii between August and October 2024. Classrooms were randomized into two study groups: the active control (HI-FLY) and intervention (HI-FLY + PSEd). Study activities were similar between groups; however, HI-FLY + PSEd also received the PSEd app. See Table 1. for an overview of study procedures.

### 2.3. Recruitment and Randomization

Recruitment was conducted using study flyers, emails, and an in-person parent night at the study school. Eligibility criteria required students to be enrolled in grades 6–8 and available to participate for the duration of the study. Guardian and youth signed consent/assent forms before participation. A participant stipend ($100 gift voucher) was provided at study completion. The goal was to recruit a minimum of 20 students per classroom, which meets the sample size requirements for a pilot study [26,27]. Classrooms were randomized into the two study groups using a sealed envelope method administered by the school principal.

### 2.4. Measures

The study activities and timelines for each study group are presented in Table 1. At Week 0, participants and guardians completed demographics surveys. Participants provided self-reported demographic data on age, grade, sex, and race. Race categories included American Indian/Alaska Native, Asian, Black/African American, Native Hawaiian or Other Pacific Islander, and White, with multiple selections allowed. If race was left blank (n = 7), race was inferred from the participant’s response to the “ethnic group/nationality” question. Missing participant grade (n = 1) or sex (n = 1) data were supplemented from the guardian-reported demographic data for the participant.

Participants also completed an adapted Mobile Device Usage Questionnaire [28,29], which was used to determine whether they owned a personal mobile phone at baseline, and whether it was a smartphone device. No additional mobile usage behavior data were analyzed. Anthropometric measurements, physical activity level (PAL) questionnaires [30], and estimated energy requirements (EER) were recorded for each participant [22,23] (Supplementary Material Section S1).

### 2.5. HI-FLY

#### 2.5.1. Study Group Overview

The HI-FLY study group received the standard 6-week HI-FLY curriculum taught by a trained community paraprofessional Nutrition Educator [31]. The HI-FLY curriculum covered six topics: Safe Food Handling, A Food Guide, Vegetables & Fruits, Rethink Your Drink, Move More Everyday, and More Meals at Home. Each session integrated key components to support nutritional education and behavior change, including: SMART goal setting (Specific, Measurable, Attainable, Realistic, Timely) [32,33], lesson worksheets, culturally tailored nutrition education videos [34], and food demonstrations with SNAP-Ed approved recipes [35].

**Figure 1:**
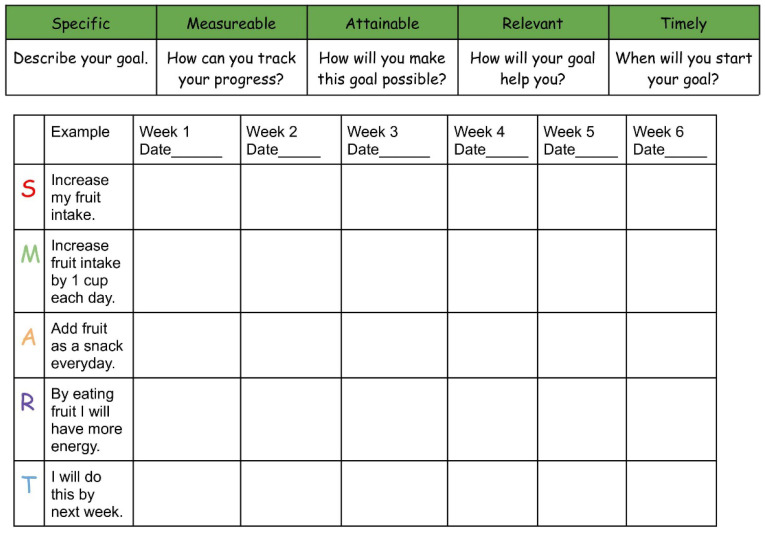
SMART Goal Setting Worksheet.

Participants set weekly SMART goals from Weeks 2 to 7 using a structured SMART goal setting worksheet (Figure 1) to support healthy behavior change [32,33]. Lesson worksheets were completed during each HI-FLY class or as homework to reinforce content understanding. Worksheet completion contributed to participant’s pass/fail HI-FLY grade.

Nutrition education videos (each ≤3 min) were developed by the research team in collaboration with SNAP-Ed. These videos focused on MyPlate food groups and interpreting Nutrition Facts label, using local foods and culturally relevant examples tailored to dietary patterns in Hawaii [7].

At the end of each HI-FLY session, the Nutrition Educator led a food demonstration using a SNAP-Ed approved recipe (e.g., Creamy Fruit & Vegetable Salad) [35]. Participants received a printed copy of each recipe and a food sample after completing their SMART goal activity.

To assess dietary change, participants completed two assigned days of written food records at Week 0 and 7, using a food record developed for the Children’s Healthy Living Program [36]. These records were used to calculate HEI-2020 scores, as detailed below.

#### 2.5.2. Youth Questionnaire

The YQ is a standard self-evaluation tool used in SNAP-Ed. The YQ consists of 15-questions completed by all students at Weeks 0 and 7 [14]. It captures self-reported behaviors related to PAL, food safety, and nutrition. For example, for nutrition-related questions, the YQ asks, “How often do you eat fruits?”, responses are rated from 1 = not very often to 5 = very often. All YQ items were scored such that higher values indicated healthier behaviors, except for the sugary drink consumption item, for which lower scores reflected improvement (Supplementary Material Section S2). All students in both classrooms completed the HI-FLY curriculum and YQ regardless of study participation, as HI-FLY was a required course by the study school.

#### 2.5.3. HI-FLY Group Dietary Assessment

The HI-FLY group completed 2 days of written food records at both Weeks 0 and 7 [36] (Supplementary Material Sections S3 and S4). These food records were completed on a Saturday and Monday to capture usual dietary intake [37,38]. Reminder text messages were sent to participants’ guardians the evening before and the morning of each logging day to reinforce adherence.

HEI-2020 scores were calculated following US Department of Agriculture and National Cancer Institute guidelines [39]. To calculate HEI-2020 scores, each food and beverage item in the written food record was coded using the Food and Nutrient Database Dietary Studies (FNDDS) nutrient values and Food Patterns Equivalent Database (FPED) food categorization (2017–2018 version for both). Total HEI-2020 scores range from 0 to 100, where higher scores represent greater adherence to the DGA 2020–2025 recommendations [40].

### 2.6. HI-FLY + PSEd

#### 2.6.1. Study Group Overview

The HI-FLY + PSEd group received the standard 6-week HI-FLY curriculum and YQ alongside the PSEd app. PSEd mobile app training occurred in Week 0 in a classroom setting. Participants practiced logging meals using plastic food models, guided by the study researcher. Brief PSEd refresher trainings (~5 min) were conducted at the beginning of each weekly class. Two researchers traveled weekly to the study school to provide PSEd technical and HI-FLY curricular support. During each class, participants used Portion Summary Screen data, a Portion Summary Worksheet, and a SMART Goals Worksheet to assist with SMART goal setting and support healthy behavior change. This group completed app-based food records at Weeks 0 and 7, allowing for pre-post evaluation of diet quality (HEI-2020 scores) as detailed below. The PSEd training, HI-FLY lessons, and SMART goal setting, were all integrated into a 75 min class period.

**Figure 2:**
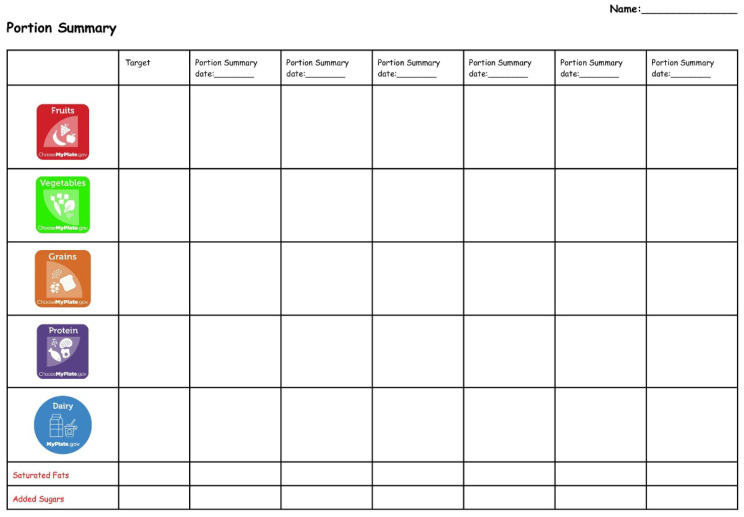
Portion Summary Worksheet.

From Weeks 2–7, the HI-FLY + PSEd participants were instructed to bring their phones to school each week to support goal setting, and phones were secured by the school office when not in use. The app’s Portion Summary Screen, which displayed detailed food intake information, was used to guide the goal-setting process. This data was transferred onto hard copy Portion Summary Worksheets (Figure 2), allowing students to visualize food group intake patterns and inform behavior change.

#### 2.6.2. PSEd App

**Figure 3:**
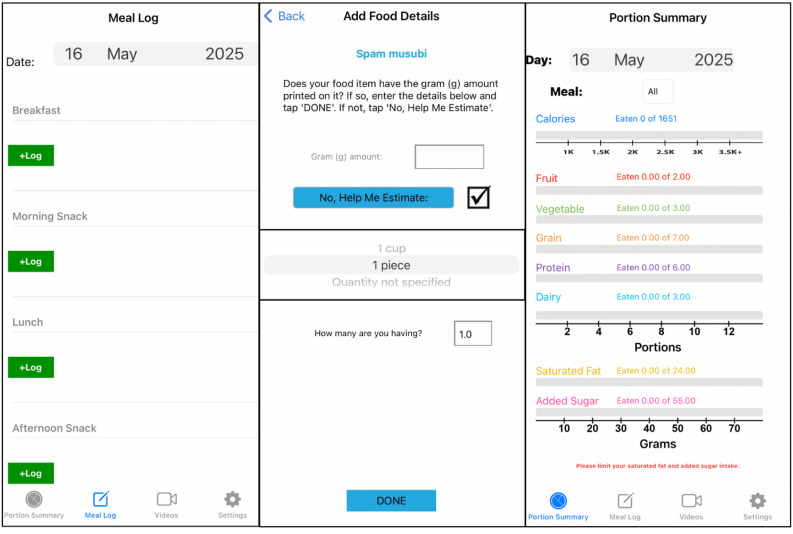
Overview of PortionSize Ed Mobile App Interface.

PSEd was installed onto each HI-FLY + PSEd participant’s study iPhone SE (3rd generation) and provided for the 7-week study. This was to ensure all participants had access to the app, and to control for type of smartphone used, as PSEd was only programmed for iOS at the time of this study. User details (age, sex, height, weight) were input into the app to produce EER and to generate personalized MyPlate food group goals [41,42]. A child-adapted version of the app was used in this pilot study, in which energy (kcal) information was removed from the Portion Summary Screen to ensure a focus on only diet quality (Figure 3). Additionally, the six culturally tailored HI-FLY nutritional education videos were embedded in the app [34].

With PSEd, users take before and after eating images and manually identify the food items and portion size estimates. PSEd contains 6556 foods from FNDDS [43] and their corresponding FPED code. In addition, the PSEd nutrient database has been customized to include 86 commonly consumed foods/recipes in Hawaii that are not found in FNDDS, which were informed by prior research from the University of Hawaii, conducted among children 2 to 13 years of age [36,44], e.g., spam musubi and loco moco (Supplementary Material Section S4). FPEDs and FNDDS nutrient data were calculated for these 86 foods and added to the PSEd database. The matching FPEDs for each food item in PSEd allows for the real-time feedback on adherence to MyPlate food group goals.

See Supplementary Materials (Supplementary Material Section S5) for details on technology set up along with app features such as the augmented reality camera, food tagging process, nutrient database, Data Capture and Analysis Platform (DCAP) [45], and the modified remote food photography method (RFPM) used to analyze food images. Full methods on the PortionSize app, which PSEd was modified from, have been published elsewhere [20,46].

#### 2.6.3. HI-FLY + PSEd Group Dietary Assessment

**Figure 4:**
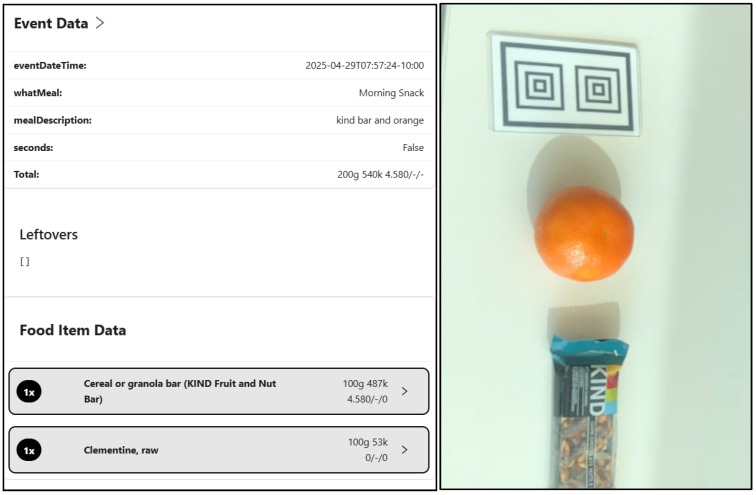
Example of a morning snack recorded by PortionSize Ed app through Data Capture and Analysis Platform (DCAP) using the reference marker.

HI-FLY + PSEd participants were required to log app-based food records on Saturday and Monday during Weeks 0 and 7, to capture usual dietary intake across a weekday and weekend day. Reminder text messages were sent to participants and their guardians the evening before and the morning of each logging day. Additionally, the PSEd app sent automatic reminders to log meals and leftovers at breakfast, lunch, and dinner. Dietary intake was assessed using the RFPM [47,48]; therefore, app-based food records could be viewed in real-time, allowing for immediate assistance and participant support from study staff if required. Trained researchers reviewed food images and the DCAP database to assess participants dietary intake as shown in Figure 4. Dietary data, with corresponding FNDDS food codes, from PSEd were exported to calculate HEI-2020 scores, as detailed above.

### 2.7. User Satisfaction Survey

At Week 7, both study groups completed a modified User Satisfaction Survey (USS), evaluating their respective food records [20,46,49]. The USS for PSEd was a 10-item survey assessing satisfaction with app-based food records in terms of ease of use, training adequacy, and overall user experience. The USS for the written food record was a 3-item survey evaluating only ease of use, food logging, and training adequacy. Quantitative responses used a 6-point Likert scale where higher scores indicated greater satisfaction (Supplementary Material Section S6).

### 2.8. Statistical Analysis

Feasibility was evaluated using two a priori outcome measures: an enrollment rate of ≥65% and an attrition rate of ≤15%. Acceptability outcomes included responses provided to the USS. Quantitative responses were summarized using means and standard deviations (SD), and independent samples *t*-tests examined group differences. All data were entered twice and cross-checked by two researchers to reduce errors introduced during data entry.

Participants were required to have at least one day of Week 0 dietary data to be included in the analysis. Daily dietary records with intakes <500 kcal or >4000 kcal were deemed implausible and excluded from analyses before averaging two-day dietary data [50]. HEI-2020 scores were calculated using National Cancer Institute SAS code (Per Day version), producing standardized, individual-level scores by daily intake [39].

To evaluate within- and between-group differences in HEI-2020 scores between Weeks 0 and 7, mixed-effects linear regression models were used [51]. The outcome was HEI-2020 score. The model included the following predictors: time (Week 0 vs. Week 7), intervention group (HI-FLY vs. HI-FLY + PSEd), and the group × time interaction. The interaction term tested whether the change in diet quality differed between groups. Baseline value of HEI-2020 score, age, sex, race, and sibling presence in other study group were included as covariates. Study group and covariates were treated as between-subjects (time-invariant) effects, while time and the interaction term were modeled as within-subjects (time-varying) effects. The same models used for the HEI-2020 scores were also run for the YQ responses. All statistical analyses were conducted using IBM SPSS (version 28.0.1, IBM Corporation, Armonk, NY, USA) or SAS (version 9.4; SAS Institute Inc., Cary, NC, USA). A significance level of *p* < 0.05 was used for all statistical tests.

## 3. Results

### 3.1. Participant Flow

**Figure 5:**
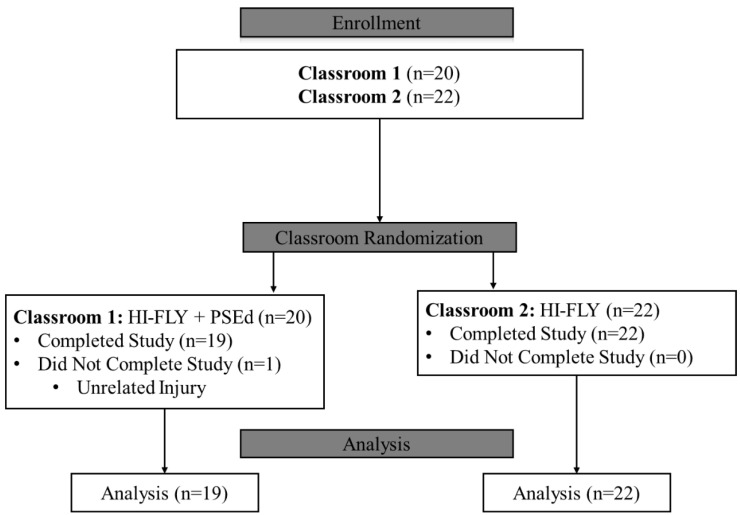
Consort Diagram detailing enrollment and randomization of study groups. HI-FLY: Hawaii—Food and Life Skills for Youth Study Group; HI-FLY + PSEd: HI-FLY combined with PortionSize Ed app Study Group.

Of the 50 participants across the two classrooms, 42 (84%) provided consent to participate. There was one dropout (2.4%) from the study due to an unrelated injury, and they were excluded from all analyses (Figure 5). HI-FLY lesson attendance was high and similar between study groups (*p* = 0.95); with mean attendance across the six lessons being 95.6% (HI-FLY + PSEd) and 95.5% (HI-FLY). All participants passed the HI-FLY course which included completing all assigned HI-FLY worksheets.

### 3.2. Participant Characteristics

Baseline demographic characteristics of participants are detailed in Table 2. The mean age of participants was 11.9 years (±0.9 SD). Nearly half of participants identified as female (48.8%; n = 20), and the majority (85.4%, n = 35) identified as at least part NHPI. No statistically significant differences were found between study groups at baseline for age, sex, race, PAL, body mass index (BMI) category, and parental education.

Four participants (9.8%) had a sibling enrolled in the opposite study group. Most participants had guardians with either some college education (41.5%; n = 17) or a bachelor’s degree (29.3%; n = 12). Most participants were classified as either underweight or healthy weight (61.0%; n = 25). A notable proportion were either living with overweight (26.8%; n = 11) or obesity (9.8%; n = 4). Of the 37 participants who completed the Mobile Device Usage Questionnaire, 61.8% (n = 25) reported owning a personal mobile phone, all of which were smartphones.

### 3.3. User Satisfaction Survey

Mean scores from HI-FLY + PSEd USS responses all exceeded the Likert scale midpoint (3.5/6), with none above 5.0 (higher scores = greater satisfaction). Mean (±SD) overall USS sub-scores were similar between groups (*p* > 0.05); 4.3 (±1.3) for HI-FLY + PSEd and 4.4 (±1.1) for HI-FLY. Also, mean USS scores were not significantly different between study groups for ease of recording food records, satisfaction for estimating serving size, and food record training.

Among HI-FLY + PSEd, the highest-rated item was “How satisfied were you with the feedback provided by PSEd regarding your meal totals?” at 4.5 (±1.3), followed by “Was it easy to use PSEd to record what you ate?” 4.4 (±1.6). The lowest-rated items were satisfaction with the PSEd “Videos” tab 3.7 (±1.2) and using the PSEd “After Photo” tab to record information about your leftover food? 4.0 (±1.3). See Table 3 for full USS quantitative results.

### 3.4. Dietary Analyses

#### 3.4.1. Healthy Eating Index-2020

Of the 19 participants in HI-FLY + PSEd, 11 provided valid food records at Week 0 and 7 at Week 7; only these records were included in the analyses (Supplementary Material Section S7). Of the 22 participants in HI-FLY, 18 participants dietary intake data were included in the analyses. A post hoc exploratory analysis was conducted to better understand the low number of participants with valid app-based food records (Supplementary Material Section S8). Among HI-FLY + PSEd participants, 88.9% (n = 8) of those who owned a personal smartphone had implausible energy intake values at both Weeks 0 and 7, compared to 28.6% (n = 2) among those who did not own a smartphone. This difference was statistically significant (*p* = 0.013). During the study, three participants in HI-FLY + PSEd reported technical difficulties (e.g., taking photos or using the Portion Summary Screen) and were provided with technical support.

Over the 7-week study, there was a downwards trend in HEI-2020 score (±SE) for HI-FLY + PSEd from 45.1 (±2.6) to 40.7 (±3.2), *p* = 0.27. Whereas among HI-FLY, there was an upwards trend in HEI-2020 scores from 41.3 (±2.0) to 45.6 (±2.0), *p* = 0.13. There was no significant change in HEI-2020 scores within or between study groups (*p* > 0.05).

#### 3.4.2. Youth Questionnaire

All participants in the study completed the YQ at Weeks 0 and 7. Significant improvements in perceived dietary behaviors were reported across both groups. A significant downtrend in sugary drink consumption was observed in both groups (*p* < 0.03), reflecting a reduction in intake (note: lower scores indicate improvement for this item). HI-FLY participants also reported an increase in making healthier food choices when eating out (*p* = 0.03). There was no significant between-group difference for either of these behaviors. HI-FLY + PSEd participants reported a significant increase in the use of nutrition facts labels to compare foods and beverages (*p* < 0.001), which was significantly greater than that of HI-FLY (*p* = 0.02). Full YQ results are presented in Table 4.

## 4. Discussion

This is the first known pilot study to assess the feasibility, acceptability, and preliminary efficacy of integrating a culturally tailored dietary assessment and nutrition education app within a SNAP-Ed school-based nutrition education program among early adolescents. This study is also unique because it was an mHealth supported intervention delivered among adolescents where ~85% identified as at least part NHPI, a historically underserved group, disproportionately affected by obesity [52]. Findings from this pilot study offer meaningful insights into the practical integration of mHealth tools into school-based healthy lifestyle interventions targeting this age group.

The study met predefined feasibility benchmarks, with a high enrollment rate (84%) and minimal dropout (2.4%), thereby supporting the intervention’s acceptability. App satisfaction ratings exceeded neutral, with ratings ranging from 3.7 for video content to 4.5 for feedback on meal totals. This suggests that while users appreciated certain features, there is still room for app improvement. USS scores were similar between the app-based and written food records (*p* > 0.05). This indicates that the app-based food record was not more burdensome to complete than the written food record. This is important as there are additional steps taken by the user when completing an app-based record to receive personalized and immediate dietary feedback.

Post hoc analyses indicated that smartphone ownership was associated with a higher proportion of implausible energy intakes among HI-FLY + PSEd participants, reflecting lower engagement due to the inconvenience of using an unfamiliar secondary device. However, it is also possible that participants without personal smartphone access, for whom using a smartphone was more novel or engaging, showed higher motivation or attentiveness in completing app-based records. While statistically significant, these comparisons are based on small subgroup sizes (e.g., n = 8 smartphone owners), and results should be interpreted cautiously. Additionally, further post hoc analyses showed that HI-FLY + PSEd participants without personal smartphone access reported higher USS scores with app-based food records (4.6/6) compared to those who owned personal smartphones (4.0/6), although this difference was not statistically significant. These exploratory findings underscore the importance of adapting PSEd delivery to be compatible with the individual’s personal smartphone where available. For instance, developing a web-based version of PSEd compatible with multiple devices could mitigate participant burden and facilitate more efficient data capture. Notably, personal smartphone ownership in the study sample (62%) was substantially lower than national estimates from 2022, which reported that 93% of youth in households earning less than $30,000/year had access to a smartphone [15]. However, as a policy at the study school, all students had access to a laptop, further supporting the need for a web-based version of the PSEd app. Also, a web app is typically more cost-effective to build and maintain than separate native iOS and Android apps.

Comparative data from recent, unpublished, laboratory-based research using the PortionSize app [20] among children aged 7–12 years in Louisiana [53]; revealed higher USS scores (6-point Likert scale) than those observed in the present [54]. Specifically, ~57% of participants in the lab-based study rated the app training as 6, compared to only 21% of HI-FLY + PSEd in the present study. Additionally, over 50% of the lab-based participants rated the app’s ease of use to capture before and after-meal photos as 5 or 6, while 42–47% of participants provided similarly high ratings in the current study. Lastly, ~70% of lab-based participants rated the app’s feedback as 5 or 6, compared to ~58% in the present study. While some differences may be influenced by study context (lab vs. school), other factors, including participant characteristics and multi-meal logging requirements, could also contribute. In the lab-based study, participants received individualized (1:1) training and were asked to log only a single meal within a controlled environment. In contrast, participants in the present study received training in a group (classroom) setting from a single trained researcher and were required to log multiple meals and to complete app-based food records in a community-based setting. Overall, lab-based findings [54] highlight the value of additional training to support user experience with the PSEd app to facilitate successful implementation in real-world contexts. Given SNAP-Ed is usually delivered in a classroom setting, training parents and teachers how to use the PSEd app may provide youth with the additional app support needed.

HEI-2020 scores did not show statistically significant changes between or within groups (*p* > 0.05). Unexpectedly, a downward trend in mean (±SE) HEI-2020 scores was observed among HI-FLY + PSEd over the 7-week period, decreasing from 45.1 (±2.6) to 40.7 (±3.2), contrasting with an upward trend in HI-FLY from 41.3 (±2.0) to 45.6 (±2.0). Interpretation of these results is restricted by the small number of HI-FLY + PSEd participants with complete and plausible records. Although the initial sample sizes met the minimum requirements for a pilot study (n = 10–15) [20,27], the final analyses for the HI-FLY + PSEd group were limited by a reduced number of complete and plausible app-based food records (n = 11 at Week 0; n = 7 at Week 7). This small sample size makes it challenging to determine preliminary efficacy for improving diet quality using HEI-2020 scores. Conversely, the YQ responses suggest potential diet-related behavioral improvements in both groups. All participants completed the YQ at both time points (n = 19 and n = 22), allowing sufficient pilot data to indicate trends. Both study groups reported a significant reduction in their intake of sugary drinks. Additionally, HI-FLY + PSEd reported a significant increase in the use of nutrition facts labels, with a significant inter-group difference. The “Nutrition Facts Label” video embedded in PSEd may have contributed to this between group difference, highlighting the importance of this multimedia educational feature. Given these YQ results, if we had the same sample size for analyses for HEI-2020 scores as we did for YQ responses, we may have seen improvements in diet quality (HEI-2020 scores) among HI-FLY + PSEd.

We note that the HI-FLY group used written food records while the HI-FLY + PSEd group used app-based records. Published literature indicates differences in sensitivity, usability, and data capture between these methods [55], which could introduce differential measurement bias and affect the internal validity of study results. To minimize potential bias, this research focuses primarily on within-group changes over time and uses the YQ, completed by both groups in the same format, to provide a consistent secondary measure for cross-group comparisons.

### Strengths and Limitations

This pilot exhibits several strengths. First, this community-based pilot was designed and delivered in partnership with SNAP-Ed Hawaii, allowing for the identification of key real-world barriers and facilitators that a lab-based trial cannot capture. This partnership with SNAP-Ed provided valuable insights into the implementation of a sustainable mHealth community-based intervention. Second, the inclusion of locally relevant foods in the nutrient database and videos added to the cultural relevance of the intervention to youth in Hawaii. Also, most of the participants were NHPI adolescents, providing a rare nutrition education platform for an underserved population that is underrepresented in nutrition and mHealth literature [56,57]. Although this may limit the generalizability of results to the broader US adolescent population, NHPI adolescents are a high-risk group for poor diet quality and obesity, making targeted and culturally relevant research into mHealth interventions crucial for addressing these risks. Although this study was conducted within the context of SNAP-Ed, the methodology and lessons learned are highly transferable to other nutrition education programs, such as the Expanded Food and Nutrition Education Program and other school-based health promotion initiatives.

This pilot study also carries several limitations which impact interpretation of the findings. First, the small number of complete and plausible app-based food records within HI-FLY + PSEd restricts the ability to determine preliminary efficacy for improving diet quality (HEI-2020 scores) and to conduct subgroup analyses, by age, sex, or SES. However, this pilot study helped to identify key barriers and facilitators for future studies to ensure accurate and complete app-based food records. One potential improvement includes engaging a community advisory board, including adolescents, to help revise and enhance the PSEd app content, such as the videos, to ensure cultural relevance, engagement, and usability. Detailed app engagement metrics (e.g., frequency of use and interactions with embedded videos) were not collected in this pilot. A larger, funded study is needed to develop back-end features to capture these metrics and better assess intervention fidelity. Second, the trial employed cluster randomization and analyzed results at the individual level, introducing potential for confounding. Key confounders were controlled for in analyses to help mitigate this risk; however, with only two classrooms, differences in the classroom environment (e.g., teacher characteristics, student composition, etc.) could account for the differences in the outcomes. Given the exploratory nature of this pilot study, the findings should be interpreted with caution and treated as preliminary. Future studies with larger numbers of classrooms and students are necessary to disentangle potential confounding and validate these preliminary findings. Conducting future cluster randomized trials with a larger number of schools would allow for analysis at both individual and group levels. Third, as the study was conducted in the same school, some participants had siblings in the other study group; however, this was controlled for in analyses. Fourth, because the HI-FLY group used written food records and the HI-FLY + PSEd group used app-based food records, differences in HEI-2020 scores between groups may reflect, in part, differences in dietary assessment methods rather than true changes in diet quality. While our analyses adjusted for study group, the potential for method-related measurement bias remains. However, both groups completed the same YQ to assess self-reported dietary behaviors, providing a consistent secondary measure across groups. Another limitation is that this study relied on self-reported outcomes, including dietary behaviors assessed via the YQ and USS ratings, which may be subject to social desirability or recall bias [58]. In addition, participants and educators were not blinded to group assignment, which could have influenced questionnaire responses, particularly in the HI-FLY + PSEd group. Future studies will aim to mitigate these limitations by incorporating a Social Desirability Scale [59]; randomizing at the school level to reduce cross-group contamination, enable masking where feasible; and report additional dietary information derived from the app-based food records (e.g., Total HEI-2020 and component scores from app-based food records). While effect cannot be confirmed from this small pilot, these results provide essential groundwork for testing clinical significance in larger controlled trials.

## 5. Conclusions

These findings contribute novel pilot data indicating that the integration of PSEd into SNAP-Ed Hawaii among early adolescents is both feasible and acceptable, with preliminary signals of efficacy that justify future hypothesis-driven trials. Although HEI-2020 scores were not significantly different between study groups, the HI-FLY + PSEd group reported significant improvements in sugary drink intake and the use of nutrition facts labels. Importantly, this trial sheds critical light on key facilitators and barriers of implementing mHealth tools into interventions in school-based settings among predominantly NHPI youth, an underserved, high-risk population. Looking forward, larger and higher-powered PSEd studies could enhance the ability to detect significant diet quality changes, while exploring sex and age-based differences. Future school-based trials should address the key barriers identified to ensure inclusivity, effectiveness, and scalability, such as enhanced PSEd training, PSEd app improvements, and developing a web-based PSEd app compatible across a wide range of technologies. With thoughtful refinement, mHealth tools like PSEd could support SNAP-Ed, and other community-based nutrition programs, to improve diet quality and health equity among youth.

## Figures and Tables

**Table 1:** Overview of Study Procedures for HI-FLY and HI-FLY + PSEd Groups.

Week	Procedure	Activities	HI-FLY	HI-FLY + PSEd
0	Recruitment and Consent/Assent	Recruitment of students from two classrooms and signed consent/assent	✓	✓
	Randomization	Classrooms randomized into study groups	✓	✓
	First Visit	Study iPhones distributed		✓
		Baseline data collection: demographics, Mobile Device Usage Questionnaire, anthropometry, physical activity level and EER	✓	✓
		Training on respective food records	✓	✓
	Baseline Food Records	HI-FLY: 2-day written food record	✓	
		PSEd: 2-day app-based food record		✓
1	HI-FLY Lesson 1	HI-FLY curriculum and Youth Questionnaire	✓	✓
	PSEd Training	PSEd training and ≥1 day app-based food record encouraged before next session		✓
2–5	HI-FLY Lesson 2–5	HI-FLY curriculum and SMART Goal Setting	✓	✓
	PSEd Training	Refresher training and ≥1 day app-based food record encouraged before next session		✓
6	HI-FLY Lesson 6	HI-FLY curriculum and Youth Questionnaire	✓	✓
7	Final Food Records	HI-FLY: 2-day written food record	✓	
		PSEd: 2-day app-based food record		✓
	Final Visit	Study iPhones collected		✓
		User Satisfaction Survey	✓	✓
		$100 gift voucher stipend provided	✓	✓

Abbreviations: HI-FLY: Hawaii—Food and Life Skills for Youth Study Group; HI-FLY + PSEd: HI-FLY combined with PortionSize Ed app Study Group. EER: Estimated Energy Requirement.

**Table 2:** Baseline Demographic Characteristics of Participants by Study Group.

Variable	Total (n= 41)	HI-FLY + PSEd (n = 19)	HIFLY (n = 22)	*p*-Value
	Mean ± SD	Mean ± SD	Mean ± SD	
Age (years)	11.9 ± 0.9	11.8 ± 0.8	11.9 ± 0.9	0.81
	n (Col%)	n (Row%)	n (Row%)	
Sex				0.28
Male	21 (51.2)	8 (38.1)	13 (61.9)	
Female	20 (48.8)	11 (55.0)	9 (45.0)	
Race				0.52
NHPI	12 (29.3)	4 (33.3)	8 (66.7)	
NHPI + 1 race	13 (31.7)	7 (53.8)	6 (46.2)	
NHPI + 2 other races	10 (24.4)	4 (40.0)	6 (60.0)	
Other ^1^	6 (14.6)	4 (66.7)	2 (33.3)	
Sibling in other study group				0.88
Yes	4 (9.8)	2 (50.0)	2 (50.0)	
No	37 (90.2)	17 (45.9)	20 (54.1)	
Parent Education				0.54
High School	8 (19.5)	3 (37.5)	5 (62.5)	
Some College	17 (41.5)	10 (58.8)	7 (41.2)	
Bachelor’s	12 (29.3)	5 (41.7)	7 (58.3)	
Postgraduate	4 (9.8)	1 (25.0)	3 (75.0)	
BMI Category				0.08
Underweight/ Healthy Weight	25 (61.0)	10 (40.0)	15 (60.0)	
Overweight	11 (26.8)	8 (72.7)	3 (27.3)	
Obese	4 (9.8)	0 (0.0)	4 (100.0)	
Missing ^2^	1 (2.4)	1 (100.0)	0 (0.0)	
Baseline PAL				0.88
Not Active	4 (9.8)	2 (50.0)	2 (50.0)	
Somewhat Active	15 (36.6)	6 (40.0)	9 (60.0)	
Very Active	21 (51.2)	10 (47.6)	11 (52.4)	
Missing ^2^	1 (2.4)	1 (100.0)	0 (0.0)	
Personal Smart-phone Ownership				0.20
Yes	25 (61.8)	9 (36.0)	16 (64.0)	
No	12 (29.3)	7 (58.3)	5 (41.7)	
Missing ^2^	4 (9.8)	3 (75.0)	1 (25.0)	

^1^ “Other” race categories included: Asian, White, and American Indian/Alaska Native and Asian. ^2^ ”Missing” participants did not compete measurement/survey question. Abbreviations: HI-FLY + PSEd: HI-FLY combined with PortionSize Ed app Study Group. HI-FLY: Hawaii—Food and Life Skills for Youth Study Group. NHPI: Native Hawaiian and Pacific Islander. PAL: Physical Activity Level.

**Table 3:** User Satisfaction Survey (USS) Results for HI-FLY and HI-FLY + PSEd Groups.

Question	HI-FLY + PSEd (n = 18) ^2^	HI-FLY (n = 22)	*p*-Value
	Mean ± SD	Mean ± SD	
Was it easy to use [x] ^1^ to record what you ate?	4.4 ± 1.6	4.4 ± 1.3	0.86
How satisfied were you with [x] for recording information about the serving size of the food you ate?	4.2 ± 1.4	4.2 ± 1.2	0.99
How much did the training help prepare you for using [x]?	4.2 ± 1.3	4.5 ± 1.1	0.47
USS Sub-Score	4.3 ± 1.3	4.4 ± 1.1	0.85
Was it easy to use PSEd to find the foods that you ate?	4.1 ± 1.5	-	-
Was it easy to use the PSEd “Before Photo” tab to record information about the food you were about to eat?	4.1 ± 1.5	-	-
Was it easy to use the PSEd “After Photo” tab to record information about your leftover food?	4.1 ± 1.5	-	-
How satisfied were you using the PSEd “Before Photo” tab to record information about the food you were about to eat?	4.2 ± 1.3	-	-
How satisfied were you using the PSEd “After Photo” tab to record information about your leftover food?	4.0 ± 1.3	-	-
How satisfied were you with the videos in the PSEd “Videos” tab?	3.7 ± 1.2	-	-
How satisfied were you with the feedback provided by PSEd regarding your meal totals	4.5 ± 1.3	-	-
USS Total Score	4.1 ± 1.2	-	-

Difference assessed using independent samples *t* tests. Responses used a 6-point Likert scale (1 = least favorable, 6 = most favorable). ^1^ “[x]” indicates study groups’ respective food recording method (written or app-based). ^2^ Missing data: HI-FLY + PSEd group (n = 1). “-” indicates not applicable. Abbreviations: HI-FLY: Hawaii—Food and Life Skills for Youth Study Group; HI-FLY + PSEd: HI-FLY combined with PortionSize Ed app Study Group.

**Table 4:** Mean Youth Questionnaire Results for HI-FLY and HI-FLY + PSEd Study Groups, and Within- and Between-Group Changes.

Variable	HI-FLY + PSEd Study Group					HI-FLY Study Group					Between Group Change
	Pre	Post	Pre	Post		Pre	Post	Pre	Post		
	n	n	YQ Score	YQ Score	*p* ^1^	n	n	YQ Score	YQ Score	*p* ^1^	*p* ^2^
Q1. Fruits	19	19	3.99	3.67	0.06	22	22	3.96	4.05	0.55	0.07
Q2. Vegetables	19	19	3.37	3.24	0.57	22	21	3.48	3.44	0.88	0.76
Q3. Sugary Drinks	19	19	3.36	2.83	0.004	22	22	3.37	3.01	0.03	0.49
Q4. Whole Grains	19	19	2.31	2.78	0.30	22	22	1.78	2.46	0.11	0.74
Q5. Healthy Choices Eating Out	18	18	2.27	2.60	0.28	22	22	2.24	2.87	0.03	0.46
Q6. Reading Nutrition Labels	19	19	2.39	3.23	0.0009	22	22	2.48	2.57	0.68	0.02
Q7. PAL Cardio	19	19	4.74	4.79	0.87	22	22	4.77	4.86	0.77	0.93
Q8. PAL Strengthen	19	18	3.11	3.49	0.32	22	21	3.02	4.39	0.0004	0.07
Q9. Choose to include PAL	19	19	3.99	3.67	0.14	22	22	3.83	3.92	0.65	0.17
Q10. Washing Hands	19	19	3.97	3.97	0.99	22	22	3.93	4.07	0.45	0.61
Q11. Washing Fruit and Vegetables	19	19	3.91	3.97	0.76	22	21	4.00	4.11	0.52	0.82
Q12. Separate Boards When Cooking	19	19	3.37	4.10	0.06	21	21	3.38	3.15	0.51	0.07
Q13. Food in refrigerator within 2 h	19	19	3.81	4.12	0.25	22	22	4.08	3.94	0.59	0.23
Q14. Comparing price of foods	19	19	3.56	3.40	0.70	22	22	3.47	3.34	0.72	0.97
Q15. Preparing homemade meals	19	19	3.95	3.95	0.99	22	22	3.63	3.82	0.48	0.63

^1^ Change in mean YQ scores within study groups for all participants. ^2^ Change in mean YQ scores between study groups for all participants. All analyses ran using mixed-effect models adjusted for covariates: Age, Sex, Race, Sibling, Baseline Variable. Note: YQ Responses are rated from 1 = not very often to 5 = very often. Q4,5,12,14,15 also include 0= no choice. Q7 and 8 range from 1= 1 day/week to 7 = 7 days/week. For the sugary drink consumption item, a lower score reflects improved behavior. Abbreviations: HI-FLY: Hawaii—Food and Life Skills for Youth; HI-FLY + PSEd: HI-FLY combined with PortionSize Ed app. PAL = Physical Activity Level.

## Data Availability

The data presented in this study are available on request from the corresponding author. The data are not publicly available due to the small sample size.

## Supplementary Materials

The following supporting information can be downloaded at: https://www.mdpi.com/article/10.3390/nu17193145/s1, Section S1: Additional Information on Anthropometric Data Collection, Physical Activity Assessment, and Estimated Energy Requirements. Section S2: Images of the 15-Question SNAP-Ed Youth Questionnaire. Section S3: Additional Details on HI-FLY Study Group Written Food Records. Section S4: Image of the Children’s Healthy Living Written Food Record. Section S5: Technical Features of the PortionSize Ed App, Including Setup, Augmented Reality Camera, Food Tagging, Nutrient Database, and Modified Remote Food Photography Method. Section S6: Images of the User Satisfaction Surveys for Both Study Groups. Section S7: Exclusion Details for HEI-2020 Analysis at Baseline (Week 0) and Final (Week 7). Section S8: Post Hoc Analysis of Smartphone Ownership, Plausibility of Recorded Energy Intake, and User Satisfaction. References [37,45,60,61,62,63,64] are cited in Supplementary materials.

## Abbreviations

BMI	Body Mass Index
DCAP	Data Capture and Analysis Platform
DGA	Dietary Guidelines for Americans
FNDDS	Food and Nutrient Database Dietary Studies
FPED	Food Patterns Equivalent Database
HEI-2020	Healthy Eating Index-2020
HI-FLY	Hawaii—Food and Lifeskills for Youth
mHealth	mobile Health
NHPI	Native Hawaiian and Pacific Islander
PAL	Physical Activity Level
PSEd	PortionSize Ed
RFPM	Remote Food Photography Method
SMART	Specific, Measurable, Attainable, Realistic, Timely
SNAP-Ed	Supplemental Nutrition Assistance Program Education
US	United States
YQ	Youth Questionnaire
