# Supplementary material for: Integration of the PortionSize Ed App into SNAP-Ed for Improving Diet Quality Among Adolescents in Hawaii: A Randomized Pilot Study
Source: Nutrients. 2025 Oct 1;17(19):3145. doi: 10.3390/nu17193145 (PMC12526172; doi:10.3390/nu17193145)
Supplement: Supplementary file 1 [file nutrients-17-03145-s001.zip › nutrients-3856384-supplementary.pdf]

Supplementary Material

Integration of the PortionSize Ed app into SNAP-Ed for Improving Diet Quality Among Adolescents in Hawai‘i: A Pilot Study

Emerald Proctor, Kiari H. L. Aveiro, Ian Pagano, Lynne R. Wilkens, Leihua Park, Leilani Spencer, Jeannie Butel, Corby K. Martin, John W. Apolzan, Rachel Novotny, John Kearney, Chloe P. Lozano

Section S1: Additional Information on Anthropometric Data Collection, Physical Activity Assessment, and Estimated Energy Requirements

Anthropometry

Participant heights and weights were taken using a stadiometer (PE-AIM-101; Perspective Enterprises) and a digital scale (Seca 876) using standard methods. Each measurement was recorded twice and averaged. If discrepancies exceeded 0.5 cm or 0.5 kg, a third measurement was taken. Anthropometric measurements for each participant were taken one at a time and away from other students. Participants faced away from the display of the digital scale to avoid any focus on body weight. Heavy clothing was removed to ensure accuracy. Participant age, sex, height, and weight were inputted into the CDC calculator to determine BMI].

Physical Activity Questionnaire And Estimated Energy Requirements

PAL for the EER calculations was determined using a physical activity questionnaire (31), which was collected at Week 0. Participants self-reported their PAL using three categories including “Not Active” (0-30min), “Somewhat Active” (30-40 min), or “Very Active” (≥40 min). Age, sex, anthropometry, and PAL data were input into the MyPlate Plan to determine participant’s EER (23, 24).

Section S2: Images of the 15-Question SNAP-Ed Youth Questionnaire

EFNEP
Expanded Food and Nutrition Education Program

6th - 12th Grade EFNEP Youth Questionnaire

Student Name or ID:__________________________ **Grade:** ______________ **Date:** _____________ ☐ **Pre** ☐ **Post**

Please pick one answer for each question. Mark the bubble with your response.

1. **How often do you eat fruits?** *Include fresh, frozen, canned and dried fruits. Do not include juice.*

   ① Not very often ② ③ Sometimes ④ ⑤ Very often

2. **How often do you eat vegetables?** *Include cooked, frozen, canned, fresh vegetables, and salads. Do not include deep-fried vegetables (such as French fries).*

   ① Not very often ② ③ Sometimes ④ ⑤ Very often

3. **How often do you drink sugary drinks like soda/pop, fruit-flavored drinks, sports drinks, energy drinks, and/or sweetened tea/coffee drinks?** *Do not include 100% fruit juice or diet soda/pop.*

   ① Not very often ② ③ Sometimes ④ ⑤ Very often

4. **When you have a choice, how often do you choose whole grains?** *Like brown rice instead of white rice, whole grain bread instead of white bread, and whole grain cereals.*

   ① Not very often ② ③ Sometimes ④ ⑤ Very often ⓪ I do not have a choice

5. **When you eat out at a restaurant or fast-food place or get take-out, how often do you make healthier choices when deciding what to eat or drink?**

   ① Not very often ② ③ Sometimes ④ ⑤ Very often ⓪ I do not eat at those places

6. **How often do you use the Nutrition Facts Label to compare packaged foods or drinks?**

   ① Not very often ② ③ Sometimes ④ ⑤ Very often

7. **In the past 7 days, how many days were you physically active enough that your heart beat fast and you were breathing hard most of the time?**

   ⓪ 0 days ① 1 days ② 2 days ③ 3 days ④ 4 days ⑤ 5 days ⑥ 6 days ⑦ 7 days

8. **During the past 7 days, on how many days did you do exercises to strengthen or tone your muscles, such as push-ups, sit-ups, or weight lifting?**

   ⓪ 0 days ① 1 days ② 2 days ③ 3 days ④ 4 days ⑤ 5 days ⑥ 6 days ⑦ 7 days

Cooperative Extension offers equal program and employment opportunities. *Y-2024* **1**

6th - 12th Grade EFNEP Youth Questionnaire

Student Name or ID:____________________ **Grade:** __________ **Date:** __________ ☐ **Pre** ☐ **Post**

Please pick one answer for each question. Mark the bubble with your response.

9. **How often do you make choices to include physical activity into your day?** *Like walking or biking instead of getting a ride, doing a few minutes of exercise, choosing technology that involves physical activity, or moving actively in your home.*

①	②	③	④	⑤
Not very often		Sometimes		Very often

10. **How often do you wash your hands with soap and running water for at least 20 seconds before making or eating food?**

①	②	③	④	⑤
Not very often		Sometimes		Very often

11. **How often do you wash fruits and vegetables before eating them?**

①	②	③	④	⑤
Not very often		Sometimes		Very often

12. **When making food, how often do you use separate cutting boards for raw meats and fresh produce?** *Also count when you wash a single cutting board with warm, soapy water when switching between these foods.*

①	②	③	④	⑤	⓪
Not very often		Sometimes		Very often	I do not make my own food

13. **When you take foods out of the refrigerator, how often do you put them back within 2 hours?**

①	②	③	④	⑤
Not very often		Sometimes		Very often

14. **How often do you compare prices of foods or drinks at the store before you buy them?**

①	②	③	④	⑤	⓪
Not very often		Sometimes		Very often	I do not buy food

15. **How often do you make your own snack or meal instead of purchasing one?**

①	②	③	④	⑤	⓪
Not very often		Sometimes		Very often	I do not make my own food

Section S3: Additional Details on HI-FLY Study Group Written Food Records

During Week 0, participants received training from the Nutrition Educator and research team on how to accurately complete these records and practiced logging an example meal [61]. Entries included information such as the start and end times of each eating occasion, specific food descriptions, preparation methods, eating locations, and any concurrent activities (e.g., watching TV or socializing). To support accuracy, participants were instructed to collect wrappers and labels of food and beverage consumed on logging days in labeled zip-lock bags. These materials were used to cross-reference written records (37). Measuring utensils (e.g., cups and spoons) were provided to assist with portion size measurements.

Section S4: Image of the Children's Healthy Living Written Food Record *(37)*

Day 1 Date: ____/_____/______ Mon Tue Wed Thu Fri Sat Sun (check one) Participant ID ____________

Month Day Year

			FOOD LOG				
	Time start	Time end	Detailed Description of Foods & Beverages	Amount	Place Prepared	Place Eaten	Other Activities While Eating
1							
2							
3							
4							
5							
6							
7							
8							
9							
10							

Section S5: Technical Features of the PortionSize Ed App, Including Setup, Augmented Reality Camera, Food Tagging, Nutrient Database, and Modified Remote Food Photography Method

PortionSize Ed Setup

Each participant received an Apple iPhone SE (3rd generation), preloaded with the PSEd app and equipped with a case, charger, and reference cardholder. Phones were restricted with no browser access and managed with a generic Apple ID. Participants had unlimited text and calls and could contact study staff for support. Prior to phone distribution, participants' characteristics were entered into the app to calculate EER. If phones malfunctioned, replacements were provided.

Augmented Reality Camera

Participants recorded meals using an augmented reality camera in combination with a fiducial marker. A credit card-sized reference marker of known dimensions was provided, featuring a visual portion size guide, adapted from MedlinePlus guidelines (e.g., "1 tablespoon = size of an ice cube") [62], to assist participants with manual portion estimation. Participants were instructed to include the marker in the frame when capturing food images to ensure accurate angle and distance, facilitating volume estimation by researchers.

Food Tagging Process

Foods were tagged using either the search function (typing food names or brands) or a hierarchical category picker (e.g., Fruits > Raw & Processed Fruit > Apple, raw). The "Help Me Estimate" tool enabled portion sizes to be recorded in familiar units (grams, fluid ounces, cups, tablespoons, slices). Drop-down portion size options were aligned with the Portions and Weights data from the Survey Food items in the FNDDS 2017–2018 database, as available on FoodData Central [63]. If a meal logging occasion was missed, participants could use the "Record from Memory" tool to enter foods manually.

PSEd Food and Nutrient Database

The PSEd app utilized a customized nutrient database including 6,556 standard FNDDS foods and 86 culturally relevant foods for Hawai'i. The additional culturally common foods were informed by prior research from the University of Hawai'i, conducted among children 2 to 13 years of age [37, 45, 64], and matched them with food data listed in the 2017-2018 FNDDS]. Where no direct FNDDS match existed, study staff created food entries using recipes and ingredient-level coding. This approach ensured nutritional accuracy and cultural appropriateness of the food records] See list below of 86 additional food items added to nutrient database:

1. andagi, 2. anpan, 3. arare, 4. banana gingerbread, 5. beef meat jun, 6. beef soup with tofu & miso, 7. bibinka, 8. bigeyed fish (akule, halalu, aji), 9. bulanglang, 10. bulgogi or pulgogi, 11. bunelos manglo, 12. chicarron, 13. chicken dinola, 14. chicken katsu, 15. chicken long rice, 16. chicken noodle casserole, 17. chigae, 18. chocolate haupia pie, 19. dashi soup, 20. egg foo yong or egg foo young, 21. ensamada, 22. fried spam, 23. furikake, 24. gau gee, 25. gon lo mein, 26. hapa rice, 27. jam bong, 28. kabocha risotto with miso, 29. kai choy, 30. kalbi ribs, 31. kalua pig, 32. kalua pork & cabbage, 33. kamaboko, 34. konbu, 35. lilikoi passion drink, 36. loco moco, 37. long john, 38. lup chong, 39. macaroni potato salad w/ mayonnaise, 40. manju, 41. meat jun sauce, 42. mochi, 43. mochi ice cream, 44. mochi w/ sweet bean paste, 45. moonfish (opah), 46. mun doo, 47. musubi, 48. nishime, 49. nori, 50. okinawan sweet potato-haupia pie, 51. okoshi, 52. oyako donburi, 53. pancit canton, 54. pao doce bread, 55. parrot fish (uhu), 56. passion-orange drink, 57. passion-orange-guava nectar, 58. pickled mango, 59. poi, 60. pork long rice, 61. pork luau, 62. portuguese bean soup, 63. postum, 64. prune mui, 65. raw opihi, 66. seasoned okara, 67. som tam, 68. somen salad w/ sauce, 69. spam musubi, 70. squaw, 71. stir-fried fish cake, 72. takuan or takuwan, 73. tang mein, 74. teppo or tekka, 75. tinola, 76. tofu lemongrass curry, 77. tom yum goong soup, 78. tonkatsu sauce, 79. tuna fish salad, 80. ume or umeboshi, 81. wakame, 82. warabi, 83. wheat noodle soup, 84. yaki soba, 85. yardlong beans, 86. yellow bean sprout namul.

Data Capture and Analysis Platform

All data captured from the PSEd app were securely transferred to the Data Capture and Analysis Platform (DCAP) (46). DCAP served as the primary platform for data storage, food image, and portion size analysis. DCAP is a secure web-based platform developed by Pennington Biomedical Research Center to store and analyze app-based food records. Each participant was assigned a unique DCAP ID linked to their PSEd account. DCAP enabled researchers to monitor meal logs, timestamps, food tags, and images in real-time.

Modified Remote Food Photography Method

The study staff first reviewed food images on DCAP to identify and estimate the amount of food consumed. In instances where meals were logged without images, e.g., if the participant used the “record from memory” flow, written descriptions were used for food identification and portion estimation. If necessary, study staff cross-checked app-based food records with images found on the study phones. If data did not transfer to DCAP, images were downloaded from the study phones and assessed using the Remote Food Photography Method (RFPM). Entries without portion estimates were assigned “quantity not specified” for analysis consistency.

Section S6: Images of the User Satisfaction Surveys for Both Study Groups

User Satisfaction Survey for HI-FLY+PSEd Study Group

	Not at all	2	3	4	5	Very much
1. Was it easy to use PortionSize Ed to record what you ate?						
2. Was it easy to use PortionSize Ed to find the foods that you ate?						
	Extremely Dissatisfied	2	3	4	5	Extremely Satisfied
3. How satisfied were you with PortionSize Ed for recording information about the serving size of food you ate?						
	Not at all	2	3	4	5	Very Much
4. How much did the training help prepare you for using PortionSize Ed?						
	Not at all	2	3	4	5	Very Much
5. Was it easy to use the PortionSize Ed "Before Photo" tab to record information about the food you were just about to eat?						
6. Was it easy to use the PortionSize Ed "After Photo" tab to record information about your leftover food?						
	Extremely Dissatisfied	2	3	4	5	Extremely Satisfied
7. How satisfied were you with using the PortionSize Ed "Before Photo" tab to record information about the food you were just about to eat?						
8. How satisfied were you with using the PortionSize Ed "After Photo" tab to record information about your leftover food?						
9. How satisfied were you with the videos in the PortionSize Ed "Videos" tab?						
10. How satisfied were you with the feedback provided by PortionSize Ed regarding your meal totals?						

Footnote: Responses used a 6-point Likert scale (column 1 = least favorable, column 6 = most favorable).

User Satisfaction Survey for HI-FLY Study Group

	Not at all	2	3	4	5	Very much
1. Was it easy to use ASA24 to record what you ate?						
2. Was it easy to use ASA24 to find the foods that you ate?						
	Extremely Dissatisfied	2	3	4	5	Extremely Satisfied
3. How satisfied were you with ASA24 for recording information about the serving size of food you ate?						

Footnote: Responses used a 6-point Likert scale (column 1 = least favorable, column 6 = most favorable).

Section S7: Exclusion Details for HEI-2020 Analysis at Baseline (Week 0) and Final (Week 7)

Group	Timepoint	Reason for Exclusion	Excluded (n)	Valid n (% of study group)
**HI-FLY+ PSEd (n=19)**	Week 0	Missing Week 0 record	4	11 (57.9)
		Implausible intake	4	
	Week 7	Missing Week 7 record	1	7 (36.8)
		Implausible intake	6	
		Invalid Week 0, valid Week 7	5	
**HI-FLY (n=22)**	Week 0	Missing Week 0 record	3	18 (81.8)
		Implausible intake	1	
	Week 7	Missing Week 7 record	2	18 (81.18)
		Invalid Week 0, valid Week 7	2	

Implausible energy intakes excluded based on predefined thresholds: <500 kcal or >4000 kcal
Baseline food record validity was required for inclusion in final HEI-2020 analysis.
Abbreviations: HI-FLY: Hawai‘i – Food and Life Skills for Youth; HI-FLY+PSEd: HI-FLY combined with PortionSize Ed app.

Section S8: Post Hoc Analysis of Smartphone Ownership, Plausibility of Recorded Energy Intake, and User Satisfaction

Post hoc Analysis: Smartphone Ownership and Energy Intake Plausibility

A Fisher's Exact Test indicated a statistically significant (p=0.035) association between personal smartphone ownership and plausibility of reported energy intake (kcal) in app-based food records at both Weeks 0 and 7. HI-FLY+PSEd participants with app-based food records that showed plausible energy intake data at both timepoints were more likely to not own a smartphone (71.4%; n=5), whereas those with implausible energy intake were more likely to own a personal smartphone (88.9%; n=8).

Table of Association Between Personal Smartphone Ownership and Plausibility of Reported Energy Intake (kcal) at Weeks 0 and 7 among the HI-FLY+PSEd Study Group

	**Plausible kcal (row %)**	**Implausible kcal (row %)**	**Total n (row %)**
Owns Smartphone	1 (11.1)	8 (88.9)	9 (100.0)
No Smartphone	5 (71.4)	2 (28.6)	7 (100.0)
Total n	6 (37.5)	10 (62.5)	16 (100.0)

Post hoc Analysis: Smartphone Ownership and User Satisfaction Survey Score

An independent samples t-test was conducted to analyze the difference in USS scores (higher=better) between HI-FLY+PSEd participants who owned a personal smartphone device at baseline and those who did not. Participants without smartphones reported higher satisfaction with PSEd food records, with a mean (±SD) score of 4.64 (±0.85), compared to 4.04 (±1.30) among those who owned smartphones. However, this difference was not statistically significant (p=0.32).
